# Referral outcomes of individuals identified at high risk of cardiovascular disease by community health workers in Bangladesh, Guatemala, Mexico, and South Africa

**DOI:** 10.3402/gha.v8.26318

**Published:** 2015-04-07

**Authors:** Naomi S. Levitt, Thandi Puoane, Catalina A. Denman, Shafika Abrahams-Gessel, Sam Surka, Carlos Mendoza, Masuma Khanam, Sartaj Alam, Thomas A. Gaziano

**Affiliations:** 1 Chronic Disease Initiative for Africa, Cape Town, South Africa; 2 Division of Endocrinology and Diabetes, Department of Medicine, University of Cape Town, Cape Town, South Africa; 3 School of Public Health, University of the Western Cape, Cape Town, South Africa; 4 Centro de Estudios en Salud y Sociedad, El Colegio de Sonora, Mexico; 5 Brigham & Women’s Hospital, Harvard School of Public Health, Harvard University, Cambridge, MA, USA; 6 Institute of Nutrition of Central America and Panama (INCAP), Ciudad de Guatemala, Guatemala; 7 School of Medicine and Public Health, University of Newcastle, Callaghan, Australia; 8 Chronic Non-Communicable Disease Unit, International Center for Diarrhoeal Disease Research, Dhaka, Bangladesh

**Keywords:** community health workers, cardiovascular risk assessment, referral outcomes, low-middle income countries, Bangladesh, Guatemala, Mexico, South Africa

## Abstract

**Background:**

We have found that community health workers (CHWs) with appropriate training are able to accurately identify people at high cardiovascular disease (CVD) risk in the community who would benefit from the introduction of preventative management, in Bangladesh, Guatemala, Mexico, and South Africa. This paper examines the attendance pattern for those individuals who were so identified and referred to a health care facility for further assessment and management.

**Design:**

Patient records from the health centres in each site were reviewed for data on diagnoses made and treatment commenced. Reasons for non-attendance were sought from participants who had not attended after being referred. Qualitative data were collected from study coordinators regarding their experiences in obtaining the records and conducting the record reviews. The perspectives of CHWs and community members, who were screened, were also obtained.

**Results:**

Thirty-seven percent (96/263) of those referred attended follow-up: 36 of 52 (69%) were urgent and 60 of 211 (28.4%) were non-urgent referrals. A diagnosis of hypertension (HTN) was made in 69% of urgent referrals and 37% of non-urgent referrals with treatment instituted in all cases. Reasons for non-attendance included limited self-perception of risk, associated costs, health system obstacles, and lack of trust in CHWs to conduct CVD risk assessments and to refer community members into the health system.

**Conclusions:**

The existing barriers to referral in the health care systems negatively impact the gains to be had through screening by training CHWs in the use of a simple risk assessment tool. The new diagnoses of HTN and commencement on treatment in those that attended referrals underscores the value of having persons at the highest risk identified in the community setting and referred to a clinic for further evaluation and treatment.

Low- and middle-income countries (LMIC) carry the highest global burden of cardiovascular diseases (CVD) and can ill-afford the considerable attendant health costs. Consequently, there is great need to establish affordable primary prevention strategies (1–3) . One such strategy is the use of a risk assessment tool that can accurately identify people at high risk of CVD who will benefit most from referral for definitive diagnoses and appropriate treatment. Risk is usually determined by calculating a risk score based on assessing a combination of risk factors, including, age, gender, tobacco use, blood pressure levels, blood cholesterol levels, diabetes or family history of CVD (4–6) . A non-laboratory-based CVD risk assessment model has been developed in response to the costs and inconvenience of the laboratory-based scores. This simplified model substitutes blood lipid levels with body mass index to calculate the absolute CVD risk score, thus making CVD risk screening far more feasible and potentially cost effective in both high- and low-resource settings (4). With the current global shortages of skilled health workers, sharing basic health promotion and disease prevention tasks with community health workers (CHWs) is gaining increasing traction and plays a crucial role in improving access to health services in under-resourced settings (5). In addition, a community-based risk assessment model has the ability to reach a larger portion of the population than a facility-based model and has been identified as key in successfully reducing and managing the rising incidence of CVD (6, 7). Early determination of CVD risk does not necessarily lead to better health outcomes unless those identified to be at risk modify their risk factors over time (6). Appropriate referral into the health system and follow-up over time is a crucial step in ensuring the success of this primary prevention strategy. High rates of attrition between CVD screening and follow-up at health facilities is common and even when those at risk are formally diagnosed and started on treatment, compliance with lifestyle changes or medication can be challenging due to the numerous financial and sociocultural barriers faced by individuals in developing countries (7, 8).

As part of a multinational study in which screening for CVD risk was conducted by CHWs in community settings, CHWs also provided those individuals identified to be at high risk with referral letters to primary health facilities for formal assessment and management (9). One of the aims of this study was to examine the immediate outcome, in terms of attendance, for high-risk individuals who were referred within existing referral pathways by a CHW to a health care facility for further assessment and management.

## Methods

The study was conducted in the four LMIC country sites of Bangladesh, Guatemala, Mexico, and South Africa. The definition of LMIC is that used by the World Health Organization (10) and all four participating sites are part of a network of centres of excellence for chronic diseases which seeks to identify problems and solutions across populations where the burden of diseases is high but the resources to address them are low.

Between 8 and 15 CHWs from each site were recruited and trained to calculate a CVD risk score using a non-laboratory-based CVD risk assessment tool. The tool uses age, sex, current smoking status, diabetes status, measured systolic blood pressure, weight and height, and a decision support chart to determine a risk score. A risk score of either <10% (low risk), 10–20% (low–moderate risk), 20–30% (moderate risk), 30–40% (moderate–high risk), or >40% (high risk) is thus calculated, where for instance, a risk score of >40% would mean that an individual had a 40% chance of having a fatal or non-fatal cardiovascular even in the next 5 years.

After demonstrating their proficiency in the above-mentioned method, each CHW opportunistically screened a minimum of 100 community members over a 4- to 6-week period in three settings at all four sites: individual homes, at community events, or at self-help groups (11). To be eligible for inclusion, community members had to be between the ages of 35 and 74 years with no reported past history of hypertension (HTN), diabetes (DM), or CVD (i.e. stroke, myocardial infarction, or angina) as these individuals were assumed to have already been part of the health system. Study participants were representative of the urban, rural, or peri-urban poor populations for each participating countries.

Two groups of participants were eligible for referral at the time of screening. 1) Urgent referrals to the closest clinic for immediate evaluation by a health professional were made for participants found to have a mean systolic blood pressure of >180 mmHg. 2) Non-urgent referrals were provided to participants with a calculated risk score of greater than 20%, and they were advised to present within 2 weeks of screening for further assessment. The referral pathways varied at each of the four country sites. In Bangladesh, dedicated study doctors were recruited to be available to assess referred community members. In Guatemala, a specific day of the week was allocated in the main primary health care centre of the community to receive referred participants. In Mexico, non-urgent referrals were made to participating health centres while urgent referrals were accepted by the general hospital. In South Africa, both urgent and non-urgent referrals were made to participating primary clinics and the field coordinator provided transport for the urgent referrals during the run-in period where four persons were found to have mean systolic blood pressure readings >180 mmHg, requiring clinical intervention. Official cooperation had been set up prior to the commencement of screenings. These ranged from three to four clinics per site. At the start of the study, permission was obtained from the relevant health authorities for study coordinators to visit the designated clinics in order to review available clinic records for the screened participants to whom a referral letter was provided.

The field team in each site accessed patient records at their respective health centres within 6 months of the initial referral. Charts were reviewed to determine whether the visit occurred, what diagnoses were made, and what treatment was initiated. Due to the suboptimal scheduling and attending of the referral visits, study coordinators were asked to implement a limited telephone contact protocol to try to reach individual participants for a total of three attempts. Each attempt had to be made at a different time of day: morning, afternoon, and evening. If the participants could not be reached using this protocol, the study coordinator noted that the referral visit attendance was ‘unknown’. Due to the resource constraints on the study, further qualitative inquiry from the participants was not possible. In addition participants who had been referred but who had not attended were contacted by the research teams to ascertain their reasons for not presenting to the respective health centres for formal evaluation and further management.

Study coordinators kept detailed notes on their findings regarding the review of medical records to determine whether referral visits were scheduled and attended. Manual coding for recurrent and divergent themes of the notes were performed by study coordinators and site investigators. Summaries of these notes were reviewed by site investigators prior to being provided to the data coordinating centre in English to allow for comparison across sites.

We conducted Pearson Chi-square tests of homogeneity to identify any patterns with respect to verifying the occurrence of referral visits, any resulting diagnoses, and any treatment provided. All data were de-identified prior to data entry and analyses. Quantitative analyses were performed using Stata 12 (12) and SAS 9.4 (13) statistical packages.

The study protocol was approved by the individual site ethics and institutional review boards (IRB), as well as the NHLBI. CHWs were trained to explain the consent forms and to answer any questions related to its contents prior to obtaining informed consent.

**Fig. 1:**
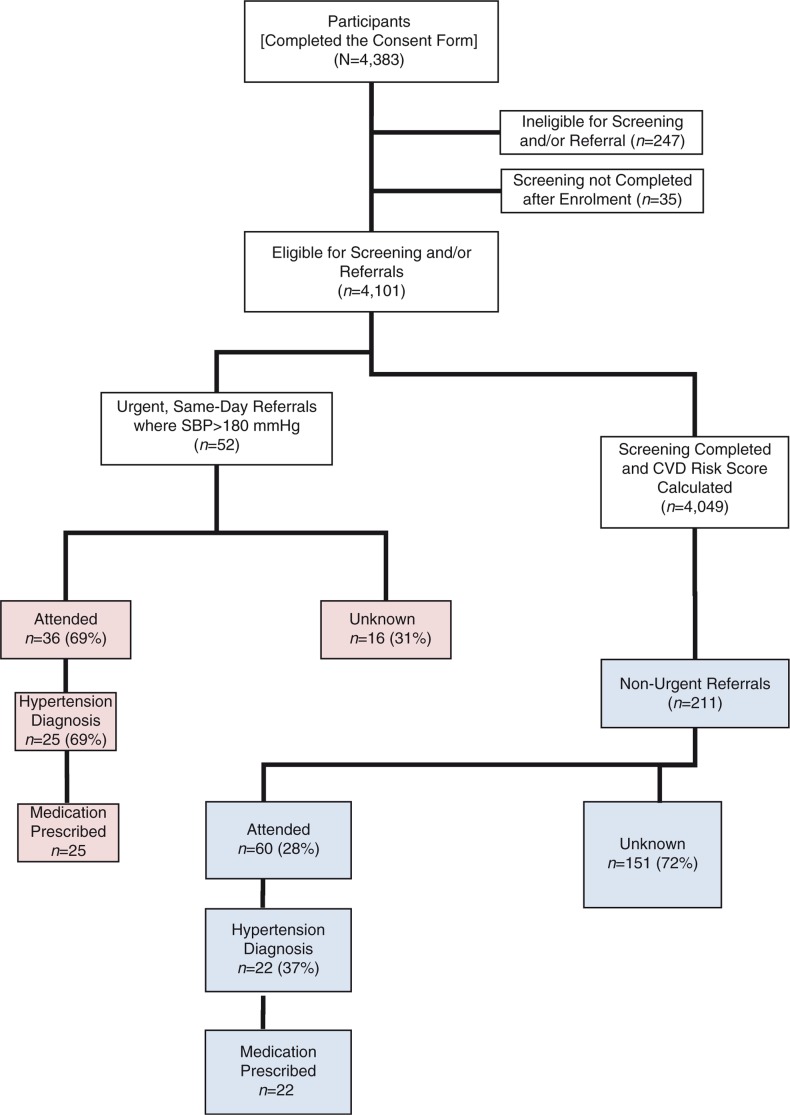
Overview of study participants.

**Fig. 2:**
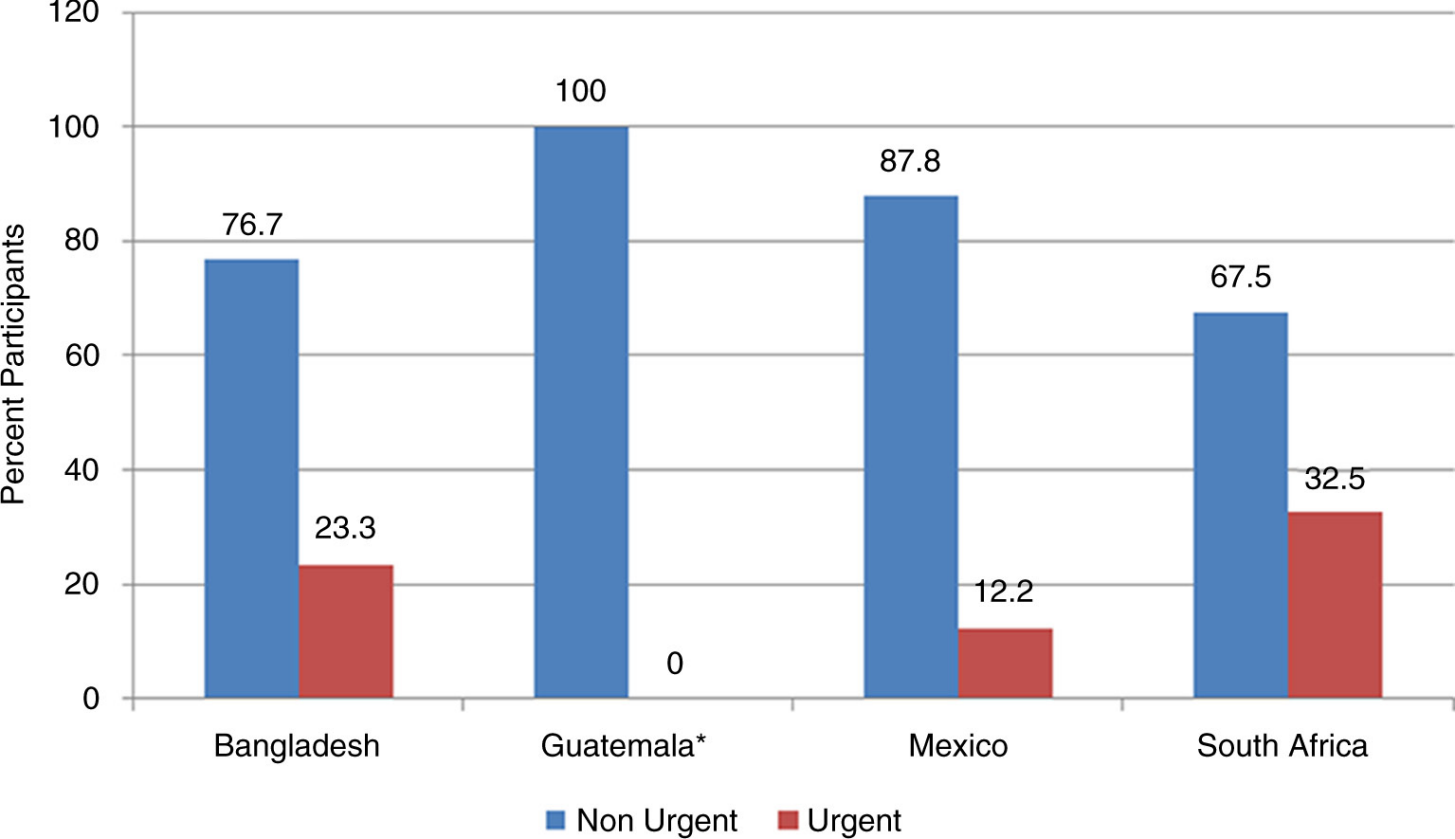
Number and type of referral by country. *In Guatemala, no subjects had an average SBP>180 mmHg which is the requirement for urgent referral.

**Fig. 3:**
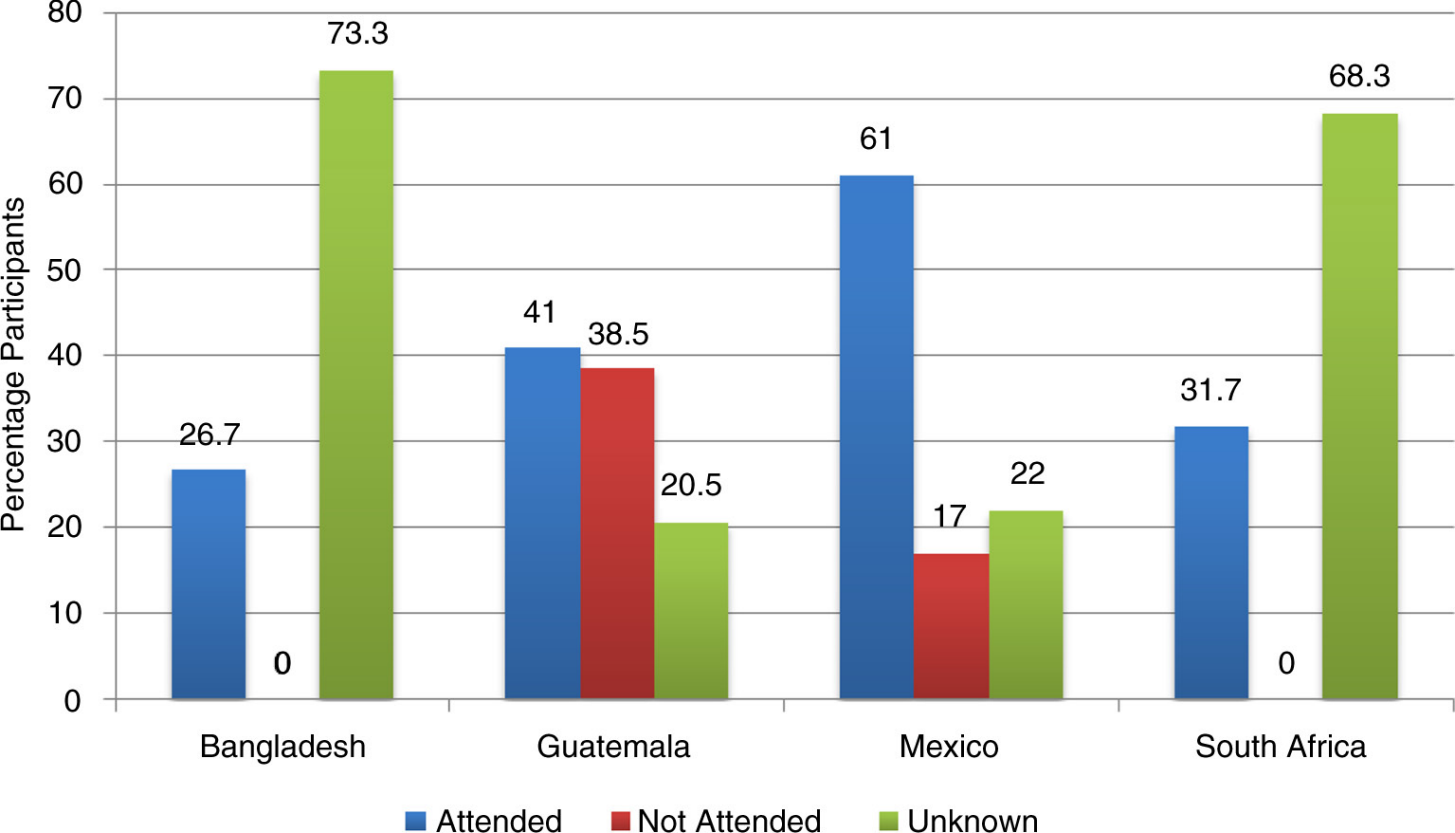
Attendance outcome by country.

## Results

### Quantitative results

Amongst the 4,383 community members who agreed to participate in this study, 247 were ineligible and 35 participants did not complete the screening process. As a result, 4,101 participants were eligible for screening or referral, of which 1,016 (24.8%) were men (range: 20.0% in Guatemala, to 28.9% in South Africa) and 3,077 (75.2%) were women (range: 71.1% in South Africa, to 80.0% in Guatemala). During screening, 52 (1.3%) were found to have an average systolic blood pressure >180 mmHg and were provided with a same-day, urgent referral letter for assessment at the nearest clinic. The remaining eligible participants (*n*=4,049) had a CVD risk score calculated by the CHWs and were stratified into one of five risk categories from low to high (Fig. 1). The distribution of the CVD risk was found to be: 77.4% low risk, 17.4% low to moderate risk, 3.7% with moderate risk, 1.1% moderate–high risk and 0.2% high risk. The risk distribution across sites was similar in all five risk categories. Participants who were at lowest risk for CVD (Risk Score from 0 to 20%) ranged from 93% (South Africa) to 97% (Mexico) of the eligible, screened population, with a mean of 95%. Those at moderate risk who were eligible for referral (Risk Score from 21 to 40%) ranged from 3% (Guatemala and Mexico) to 7% (South Africa), with a mean of 5%. The proportion of persons who were eligible for urgent referral by being at highest risk (Risk Score >40%) ranged from 0% (Guatemala) to 0.3% (South Africa).

Overall 263 of 4,101 (6.4%) participants were referred. Of these 52 (19.8%) were urgent and 211 (80.2%) non-urgent referrals (Fig. 2). Amongst all the referrals, we were able to verify that 96 (36.5%) scheduled and attended a visit at the local clinic, of which 36 of 52 (69%) were by those given urgent referrals and 60 of 211 (28.4%) by those given non-urgent referrals (Fig. 3). Those with urgent referrals scheduled and attended a visit at the clinic at a greater level (69%) compared to those who were provided with a non-urgent referral (28%) (*p*<0.001; Pearson *χ* ^2^=25.6; 1 df). Additionally, at all these verified visits, 69.4% of the urgently referred persons were likely to receive a diagnosis of HTN, compared to the 37% of non-urgently referred persons (*p*<0.008; Pearson *χ* ^2^=7.1; 1 df). HTN was the only diagnosis received by all those confirmed to have attended referral visits and 100% of those diagnosed received a prescription for anti-hypertensive medication.

### Qualitative findings

A number of themes emerged from assessing the experiences of both the CHWs and the community members being screened for CVD that provide insights into the low attendance following referral as well as into the challenges in verifying follow-up data. The findings were remarkably consistent across the four country sites (Table 1).

**Table 1:** Reasons for low attendance following referral and challenges identified in verifying visits at clinics

Theme	Description
Reasons	
Risk perception	A common problem across all four sites was the disbelief of the individuals screened that their CVD level of risk was high and that a referral visit with a health professional was necessary. The lack of symptoms made the referral seem unnecessary.
Influence of traditional versus Western medical care	Many individuals deferred to their beliefs in traditional medicine over the screening assessment, which reduced the rate of follow-up at Western medical facilities.
Lack of trust in the role of CHWs in screening for CVD	Individuals were unaccustomed to CHWs performing CVD risk assessment as well as making referrals to health facilities. Their perceptions of the roles of CHWs were limited to dispensing of medication and/or provision of health education.
Acceptance of referrals made by CHWs by health professionals	The authority of the CHW to refer persons at risk to the clinics was disputed at almost all the sites. CHWs were not regarded as qualified to make referrals of the type provided in the study, by both clinical and clerical staff at the clinics. The ability of the CHWs to assess CVD risk was also perceived as a threat by the health professionals.
Fear of reprimand and the lack of support from health facilities.	Individuals did not wish to be ‘scolded’ by health personnel for not seeking help sooner despite not knowing they were at risk of CVD.
Communication barriers	Individuals that spoke different languages to the health professionals at the referring facilities anticipated difficulties in communicating and did not see the value in attending the referral visit.
Opportunity cost of attending health facilities	Individuals that were employed identified the opportunity costs related to attending clinics for the referral visits as being inhibitory.
Cost of medication	Individuals referred for formal diagnosis and treatment were not always guaranteed free access to medication. In Guatemala, for instance, referred participants were discouraged from attending due to the incurred expense of accessing medicine.
Travel cost of attending health facilities	The travel costs associated with attending a referral visit prevented some individuals from attending the referral visit.
Challenges	
Access to health facilities and patient medical records	While the individual sites had arranged for referral visits to take place at individual health centres with clinic directors, this permission did not always translate to administrators at various levels at these clinics accommodating the study coordinators’ attempts to verify the visits.
Patient health records could not be found	In two of the countries, patient records could not be found due to a lack of systematic re-filing of folders. Single paper records were often misplaced between different programs of care.

## Discussion

These findings demonstrate that when people are opportunistically screened in the community by CHWs and identified as being at a high risk of developing CVD, only a minority (37%) schedule and attend the local health facility after being referred for further evaluation. Where referral visits were verified, there was significantly more follow-through on the part of participants who were provided with urgent referrals (69%) compared to those provided with non-urgent referrals (28%). In addition, a new diagnosis of HTN was made in over two thirds (69%) of urgent referrals and 37% of non-urgent referrals; with 100% of these cases having treatment initiated.

Higher attendance rates from CHW-initiated referrals have been described in studies conducted in other LMICs with attendance rates ranging from 58 to 93% (12–16) . These primarily involved CHWs identifying and referring ailing paediatric patients from a community setting into the health system. Predictors of referral compliance included being clinically sick and receiving reminder visits from CHWs (13). In a study done in Ecuador evaluating referral adherence using the Integrated Management of Childhood Illness (IMCI) approach in the community, the attendance rate was found to be 58%. In this study, two factors relating to CHW actions, in providing a referral letter and in making urgent referral, reduced the risk of not attending from 96% to 19%. In Uganda the difference in urgent and non-urgent referral attendance rates within a community-based malaria treatment programme was found to be less than 10% (93% for urgent referrals and 84% for non-urgent referrals) with a high overall attendance rate of 87% (12). In a randomised control trial done in a low-income urban community site in the United States, the referral attendance rates of individuals identified with high blood pressure were compared using enhanced CHW-supported referrals with usual referrals. The attendance rate for the enhanced CHW referral intervention was 65.1% compared to 46.7% in the usual-care group (17). While these findings suggest that higher attendance rates of CHW-initiated referrals are possible, it may require additional CHW referral support (such as providing reminders and follow-up visits) in instances when asymptomatic patients are targeted compared to those that are more acutely unwell.

The qualitative data that were consistent across all settings expands on the factors that inhibited scheduling and attending visits across the four sites and include the associated costs, opportunity costs, health system barriers, and paradigms of risk perception. The latter being a particularly important consideration when designing interventions that aim to bring asymptomatic patients into the health system.

Furthermore, the disputed authority of CHWs to conduct CVD risk assessments and to refer community members into the health system was also identified as an obstacle. CHWs are seen as a threat by existing health professionals and are not accepted as being adequately qualified to refer persons at high risk for further assessment at the health clinics. While this may be the case when CHWs refer asymptomatic patients, there is no evidence of resistance to CHW referrals when sick patients are identified in the community and referred into the health system (13, 15).

A number of behaviour theories have been developed to explain the failure of people to participate in health-screening activities. The Health Belief Model is one example, which suggests that *people’s beliefs about health problems, perceived benefits and barriers to action, and self-efficacy explain engagement (or lack of engagement) in health-promoting behaviour* and where a cue to action is also required to trigger the health-promoting behaviour (18). Our findings suggest that providing a referral that is urgent is more likely to lead to a health-promoting behaviour (in attending the referral) and may therefore be a more effective cue to action, compared to providing a non-urgent referral to someone that is asymptomatic.

A limitation of this study was the inability to confirm attendance after referral in the majority of cases, due to inherent health system challenges such as lack of access to records, inability to find records, and incomplete records as well as challenges in directly contacting mobile community members. This was a study done in real-world settings where our findings underscore that current primary care practices in these settings *did not* allow for efficient tracking of patients who are referred. For this reason, it is difficult to comment on the different rates of attendance across the four settings. The design choice to not alter existing referral pathways beyond what we did was intended to provide evidence for the anecdotal impression that existing referral pathways at the sites were not effective in ensuring that those identified at risk of CVD are adequately bought into the health system.

There are a number of avenues for future research. One is in understanding how community members perceive risk and why they choose to attend clinic when identified at high risk when referred by CHWs. In addition, work is also required in testing new models of referral pathways, such as linking CHWs to nurses or larger care teams, or creating dedicated appointment slots at health centres for individuals at high risk of CVD.

## Conclusion

The existing barriers to referral in the health care systems negatively impact the gains to be had through screening by training CHWs in the use of a simple risk assessment tool. The new diagnoses of HTN and commencement on treatment in those that attended referrals underscores the value of having persons at the highest risk identified in the community setting and referred to a clinic for further evaluation and treatment. If the referral mechanisms and medical record access can be improved, these would result in even further gains in diagnoses and treatment resulting from CHWs conducting opportunistic community-based screenings.
